# A Subject-Specific Surface EMG Model for Estimating L4/L5 Compressive Loading

**DOI:** 10.3390/bioengineering13010070

**Published:** 2026-01-08

**Authors:** Pablo J. Dopico, Audrey Zucker-Levin, Kunal Singal, William M. Mihalko

**Affiliations:** 1 University of Tennessee Health Science Center–Campbell Clinic Department of Orthopaedic Surgery and Biomedical Engineering, Memphis, TN 38104, USA; dopicophd@outlook.com; 2 School of Rehabilitation Science, College of Medicine, University of Saskatchewan, Saskatoon, SK S7N 5A2, Canada; audrey.zuckerlevin@usask.ca; 3 Physical Therapy, College of Rehabilitation Sciences, University of St. Augustine for Health Sciences, Austin, TX 78679, USA; ksinghal@usa.edu

**Keywords:** low back pain, electromyography, compressive loading

## Abstract

Low back pain (LBP) is a common cause of activity limitation in individuals that can result in socioeconomic costs up to $200 billion per year. Most cases of LBP lack a known underlying pathology. The L4/L5 motion segment is the most impaired lumbar segment, likely due to high load-bearing function. The ability to model L4/L5 compressive loading from surface electromyography (sEMG) data during dynamic activity may add to the understanding of LBP. Eight volunteers with no history of LBP participated in this study. Muscle activity of the erector spinae, rectus abdominus, and external obliques were recorded by a wireless EMG system (Trigno, Delsys, Natick, MA, USA) during a straight-leg stoop-to-stand task. L4/L5 compressive loading was estimated using a subject-specific sEMG model and validated by comparison with an AnyBody model and publicly available data from OrthoLoad. A specific trendline showed a significant decrease in percent error of estimated force for all muscles. Significantly lower impulse values were estimated by the AnyBody model than the sEMG subject-specific model (*p* = 0.007). Although our sEMG model was subject to high variability, loading values largely remained within those reported in the literature. Significant variation was found comparing the sEMG model with the AnyBody model, which may validate continued development and testing of personalized measurements of L4/L5 loading.

## 1. Introduction

Low back pain (LBP) is the most common cause of activity limitation in individuals under the age of 45 [1]. Due to its high prevalence, LBP results in the loss of 149 million workdays and socioeconomic costs ranging from $100 to $200 billion a year [2]. For these reasons, there is a significant interest in understanding and treating LBP. Specific LBP has an underlying pathology identified as the source of pain [3]. Nonspecific LBP has no identified underlying pathology [3,4], is more common, and accounts for 85% of patients suffering from LBP [4]. While pathologies causing LBP are difficult to identify, multiple risk factors including large and cumulative loading of the spine have been linked to increased incidence [5,6]. Likewise, increased body mass index (BMI), frequent lifting, and increased periods of sitting all increase LBP incidence due to a resulting increase in intervertebral disk (IVD) loading [7,8,9,10].

Another risk factor for individuals with LBP is decreased hamstring extensibility (tight hamstrings) [11]. Decreased hamstring extensibility tilts the pelvis posteriorly which decreases lumbar lordosis (LL) [12,13,14]. Reduction in LL increases compressive IVD loading in the spine [15], which suggests that tight hamstrings potentially alter loading patterns and increase LBP. Hamstring stretching is among the interventions prescribed by physical therapists to decrease LBP, but a direct link between hamstring extensibility and IVD loading has not been established [16]. While increased IVD loading is associated with LBP, it is difficult to measure IVD loading directly. Therefore, an indirect model is needed to accurately estimate compressive loading on the spine during dynamic activity.

The use of surface electromyography (sEMG) has been suggested previously as a noninvasive method to estimate L4/L5 compressive loading at physiologically appropriate values [17,18]. Early EMG models by Granata et al. [17] estimated muscle forces from EMG signals by using a single linear gain value; compressive loading was then calculated by anthropometric data and the sum of muscle forces. More recent EMG models have been hybridized with inverse dynamic models but have continued to use a single gain value for all subjects and muscles, establishing a linear relationship between muscle force and muscle activity [18,19,20]. These models typically have reported compressive loading values either within or above previous physiological loading values [18,19,20].

Muscle activity, as measured by sEMG, is known to vary with electrode placement and muscle strength [21]. Subject-specific factors such as muscle size, muscle fatigue, and proportion of fast-twitch muscle fibers impact muscle force production [21,22,23]. Previous studies showed that general relationships of muscle force to normalized sEMG could vary by as much as 40% of maximum sEMG for the same normalized moment among individuals [24]. This variability suggests that use of a single linear gain value for analysis of sEMG data in the calculation of L4/L5 compressive loading may not be accurate.

Additionally, the linearity of the muscle activity-to-force relationship is not well-understood. Previous studies have found a nonlinear relationship between muscle force and sEMG activity for abdominal muscles and a linear relationship for trunk extensor muscles during isometric body tilt experiments when not accounting for antagonist muscle activity [25,26]. During isometric sitting tasks, Brown et al. [27] observed that the flexor agonist moment-to-sEMG relationship became linear and the extensor agonist moment-to-sEMG relationship became slightly curved when accounting for antagonist muscle activity [24,28].

Previous studies focused solely on static, isometric contractions. However, muscle force activity varies with both fiber length and contraction velocity [29,30], revealing that these isometric relationships may not be applicable to dynamic activities involving lumbar flexion and extension. Different flexion and extension speeds can affect the relationship between muscle force and sEMG activity, resulting in inaccurate sEMG-to-force relationships from models solely based on isometric activity. Other more recent reports have looked at L5/S1 forces with bending and twisting tasks with EMG recording of erector spinae and abdominal oblique musculature [31]. They used maximal contraction of muscles to normalize EMG signal output. With an 11 kg weight and a knee-to-waist and twistng task, they reported loads of six to ten times body weight using an OpenSIM 3.3 (SimTK, Stanford, CA, USA) and MATLAB 2015a (MathWorks, Natick, MA, USA) script model.

In this study, we developed a subject-specific surface EMG model for measuring L4/L5 compressive loads during dynamic activity, mimicking real life lumbar flexion and extension tasks, comparing our personalized EMG model with two available models: a modified AnyBody model and publicly available OrthoLoad data. We quantified differences in the relationship strength between muscle force and sEMG activity due to differences in specificity (subject-specific vs. general) and linearity (linear vs. logarithmic). Our approach is unique compared to other approaches, using existing available model platforms.

We aimed to investigate the importance of patient specificity and nonlinear muscle force-to-sEMG relationships and apply them to an sEMG model for estimating lumbar loading. We hypothesize that subject-specific sEMG models utilizing nonlinear muscle force-to-sEMG relationships will result in significantly stronger relationships between muscular force and sEMG activity.

## 2. Materials and Methods

All testing was performed in the University of Tennessee Health Science Center (UTHSC) Movement Science Lab. Testing for this project made use of ten optoelectronic cameras (Qualisys, Gothenburg, Sweden), two force plates (AMTI, Watertown, MA, USA), an isokinetic dynamometer (Biodex, Shirley, NY, USA), and the Trigno Wireless EMG system (Delsys, Natick, MA, USA).

After institutional review board approval, informed consent was obtained from 10 participants (25.2 y ± 3.2) with no history of LBP or back injury. These participants all had tight hamstrings, as defined by a popliteal angle greater than 30 degrees when measured by the passive knee extension (PKE) test. Inclusion criteria for the study included the following: age under 35 years; no history of low back pain or injury; tight hamstrings as measured on the PKE test. We excluded patients under the age of 18 or over 35, and any subject with a history of low back pain or trauma in the past. There were no medical comorbidities for any participants in the study, and all participants were male subjects who were not obese (BMI < 30) with an average BMI 25.2 ± 0.8.

**Figure 1:**
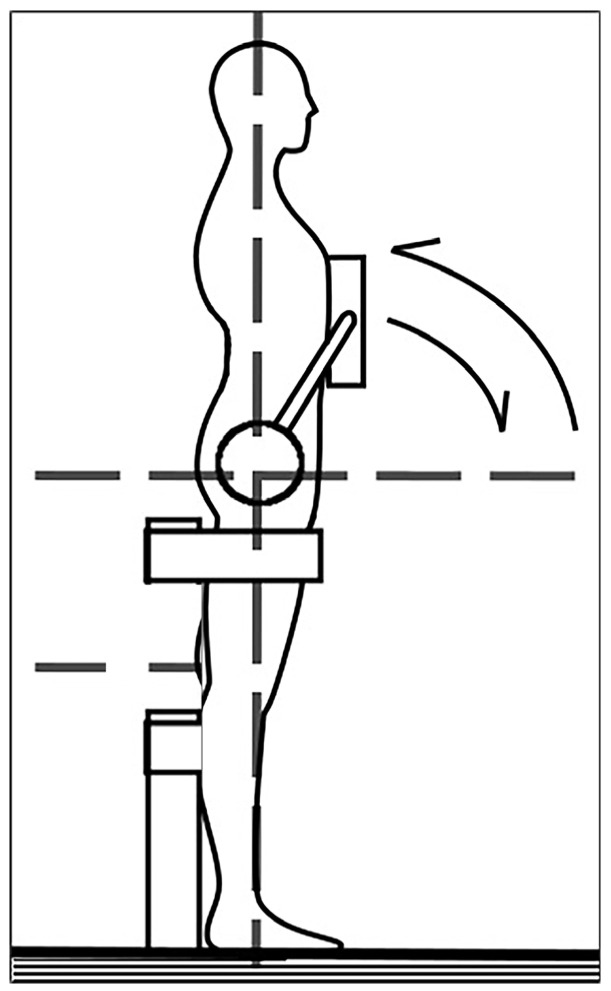
Custom dynamometer arm. Subjects were strapped from the legs down to separate torso movement from effort due to the legs. Major dashed lines indicate the sagittal and horizontal plane intersection with the axle of the rotation arm.

Surface electromyography electrodes were placed bilaterally on the erector spinae (ES), rectus abdominus (RA), and external obliques (EOs). One electrode was reserved as a timestamp to synchronize isokinetic data with sEMG data. Fifty-three reflective markers were placed on anatomical landmarks of the torso and lower extremities to track kinematic activity. To identify sEMG offset, subjects were asked to lay supine and relax for a period of 5 s while baseline sEMG signal was recorded. Voluntary contraction trials (VCTs) were performed for each subject using an isokinetic dynamometer modified with a custom arm (Figure 1).

**Figure 2:**
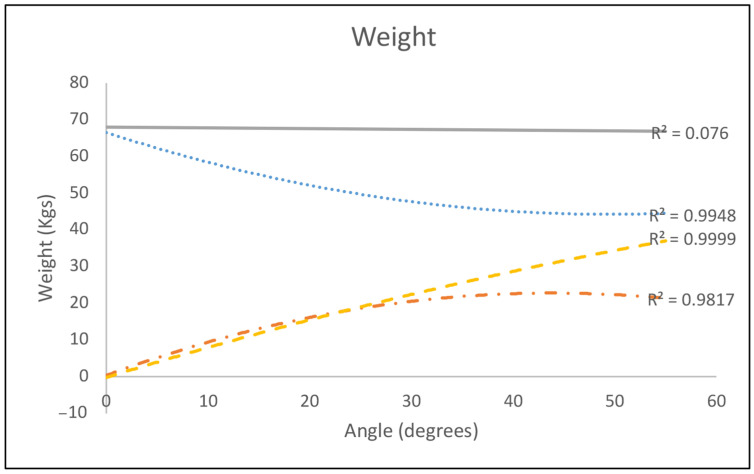
Contribution of torso weight to dynamometer torque. The body weight supported by the dynamometer (dotted dashed) and the weight accounted for by a scale (dotted) add to a constant total body weight (line). A mathematical assumption of torso weight times sin of the body angle (dashed) does not match up with the body weight supported by the dynamometer, indicating the importance of actually measuring the torso weight on the dynamometer for various angles.

The VCT consisted of three cycles of concentric trunk flexion and extension. A total of six VCTs were performed for each subject, with three each at 30 degrees per second (deg/s) and 60 deg/s of flexion extension. One trial of both 30 deg/s and 60 deg/s were performed at maximum effort while the remaining two trials were performed at varying levels of effort for each cycle. The moment induced on the custom arm was measured by the dynamometer. To adjust for effect of torso mass on the torque, a calibration curve of torque versus lever arm angle was calculated for each subject while the subject was relaxed (Figure 2).

Participants performed two 30 s bouts of the straight-leg stoop-to-stand (SLSS) tasks with each foot on one of two force plates. Participants maintained their knees in extension while repeatedly lifting an 8 kg medicine ball, holding it while upright for 2 s and then replacing the ball. Kinematic data were gathered during this time from the reflective markers placed on the subject. A single marker was placed on the medicine ball to track its position.

### 2.1. Building a Surface EMG-to-Force Relationship

Data analysis was completed using Visual3D (C-Motion, Germantown, MD, USA) and MATLAB (MathWorks, Natick, MA, USA). Visual3D was used to measure torso depth, width, and leg length as well as muscle cross-sectional areas and moment arms from each standing trial. Torso depth was defined by the distance from the sacrum to sternum in sagittal direction. Torso width was defined by the distance between iliac crest markers. Finally, leg length was defined by the vertical distance between the iliac crest and calcaneus marker. Muscle cross-sectional areas and moment arms were estimated from these measurements via anthropometric values determined by Granata et al. [17]. Since all VCTs were performed solely in the sagittal plane, bilateral muscle forces were considered equal.

MATLAB was used to build a muscle force-to-sEMG relationship for the ES, RA, and EO of each subject. Rest trial sEMG signals were subtracted from sEMG signals during VCTs to remove DC offset and a root mean square (RMS) filter with a window width of 51 was used to remove signal noise related to movement.

For both flexion and extension, the external moments (body weight, dynamometer resistance) around the L4/L5 joint were balanced by the internal muscle forces. The external moment measured by the isokinetic dynamometer during extension was attributed solely to the ES while the internal moments during flexion included both the EO and RA. Therefore, the moment balance for extension and flexion, respectively, are as follows:

$$\frac{M_{B}^{*}}{d_{L}}=F_{ES}*d_{ES}*\cos\theta_{ES}$$

$$\frac{M_{B}^{*}}{d_{L}}={(F}_{RA}*d_{RA}*\cos\theta_{RA})+{(F}_{EO}*d_{EO}*\cos45{}^{\circ})$$

where M_B_* is the moment measured by the dynamometer corrected for torso weight effects on the dynamometer during flexion or extension, respectively; *d* is the moment arm for the lever arm and muscles; *F* is the muscle force. The RA muscle was assumed to cross the same plane as the L4/L5 vertebrae at an angle of 45 degrees. The ES and RA angles were estimated from the muscle moment arm and lever arm. The ratio of cross-sectional areas for the EO and RA was used to fully balance the flexion moments. Once all forces were estimated, they were plotted with concurrent sEMG values for each muscle to produce subject-specific muscle force-to-sEMG relationships. Muscle forces and concurrent sEMG values were normalized by their maximum muscle force and sEMG values, respectively, and plotted for all subjects to construct a general relationship between muscle force and sEMG activity for each muscle.

General-versus-specific muscle force-to-sEMG relationships, different contraction velocities, and linear-versus-logarithmic relationships were compared before muscle force-to-sEMG relationships were used to calculate L4/L5 compressive loading.

### 2.2. Estimating Lumbar Loads

Visual3D was used to build an sEMG model to calculate L4/L5 compressive loading. Inputs to Visual3D included the subject’s body mass, height, and subject-specific muscle force-to-sEMG relationships for the right erector spinae (RES), left erector spinae (LES), right rectus abdominus (RRA), left rectus abdominus (LRA), right external oblique (REO), and left external oblique (LEO) muscles. Both resting and SLSS trials were imported into Visual3D.

Muscle forces were calculated for SLSS trials by inputting measured sEMG signals into the subject-specific muscle force-to-sEMG relationships. Any sEMG signals below the signal measured during rest were treated as zero muscle force. Linear force-to-sEMG relationships were used for the ES muscles while logarithmic relationships were used for the EO and RA muscles. Due to the similarities in contraction velocity with SLSS, relationships from 60 deg/sec VCTs were used to calculate muscle forces.

Once muscle force values were calculated for the trial, the compressive loading on the L4/L5 joint (F_J_) was calculated by balancing the vertical forces ($\sum F_{Z})$ acting upon the joint.

$$\sum F_{Z}={(F}_{UBW}*\cos\theta_{UBW})-F_{J}+\sum {(F}_{M}*\sin\theta_{M})$$

where *F_UBW_* is the weight of the upper body (calculated as 67% of total body weight) and *θ_UBW_* is the angle the torso made with the vertical measured from marker data.

### 2.3. AnyBody Model

A rigid body dynamic AnyBody model (MoCap LowerBody, v 6.0.5.4279, Anybody Technology, Aalbord, Denmark) was modified to work with the marker set used within the lab. This model included the legs, pelvis, torso, skull, and multiple muscles required to balance moments around joints. The arms were not included. A model optimization was performed to fit the skeletal model dimensions to our marker set before an inverse dynamic analysis was run to calculate compressive loading on the L4/L5 joint.

### 2.4. OrthoLoad Model

Three subjects from OrthoLoad’s public database were used as a source of in vivo compressive load data for the same SLSS task, except the weight was 7 kg. The data were utilized as another comparison with the AnyBody and sEMG model, resulting in load values. The OrthoLoad subjects were similar in weight (633 N + 70) compared to our subjects (677 N + 116).

### 2.5. Statistics

One subject had a critical marker missing for the AnyBody analysis, resulting in a total of eight subjects included in AnyBody analysis of compressive loading. Errors in data collection resulted in the inability of the EMG model to analyze two subjects, resulting in seven participants being available for analysis by the EMG model.

Percent error differences for relationship linearity (linear vs. logarithmic), specificity (general vs. specific), and contraction speed (30 deg/s vs. 60 deg/s) were compared using a four-way repeated measures analysis of variance (ANOVA) for each muscle in SPSS (version 23, International Business Machines, Armonk, NY, USA). A *p*-value of 0.05 was considered significant.

For the sEMG model, AnyBody model, and OrthoLoad data, impulse values were calculated by integrating compressive loading throughout a lift cycle and normalizing by each subject’s body weight. Lifting cycles were defined to have started when the participant first lifted the weight and ended when the weight was replaced on the ground. The average impulse value was then found for each participant. Two-tailed paired *t*-tests were performed between the AnyBody and the EMG model (*n* = 7) to detect differences between reported loading values. Normality of the AnyBody (*p* = 0.81) and the EMG (*p* = 0.25) were confirmed by the Shapiro–Wilk test.

## 3. Results

A summary of error results for muscle force-to-sEMG relationships can be found in Table 1.

For all muscles, the use of a specific trendline showed a significant decrease in percent error of the estimated muscle force. No muscle except the LRA (*p* = 0.023) demonstrated a significant difference in percent error when comparing linear and logarithmic muscle force-to-eEMG relationships. When generating the LL model, it was decided to use the muscle force-to-sEMG relationship that resulted in the lowest percent error regardless of statistical significance. No differences were found in the percent error due to the effects of contraction speed or time of measurement (how long it took the subject to complete the lifting trial) differences between subjects.

**Figure 3:**
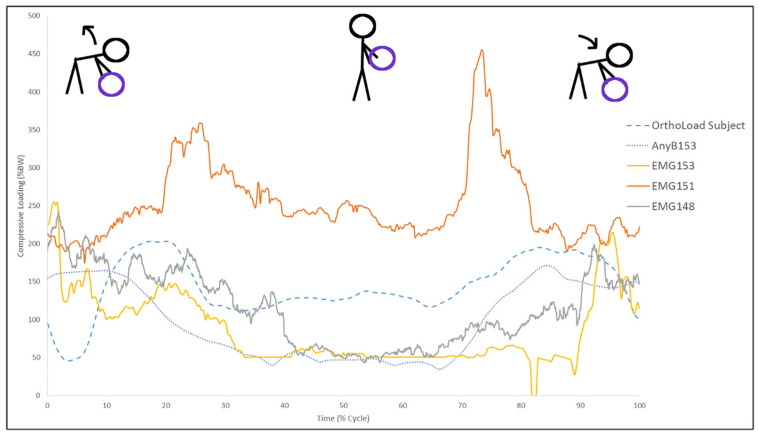
Lumbar loading of all three models. The graph is normalized with the *x* axis as the percentage of the performed task completed and the *y* axis as the percentage of bodyweight (BW) to adequately compare the three models. The figures above the chart visualize which step of the lifting task the Time corresponds to, beginning with ball lift and ending with replacement. Loading of one OrthoLoad subject and three subjects included in this study. In two cases, the loading patterns of the electromyography (EMG) model (subject 148 and 153) appear similar to AnyBody. In one case, high EMG signal resulted in abnormally high loading estimates (Subject 151). Impulse values were calculated as the areas underneath these curves.

Similar loading patterns of maximum and minimum compression values were observed between both models and OrthoLoad (Figure 3).

Loading values were lowest after the subject finished lifting and stood upright while maximum values were observed shortly after lifting began or right before replacing the weight. The range between maximum and minimum values for both models was larger than observed in the OrthoLoad data. Significantly lower impulse values were estimated by the AnyBody model compared to the sEMG model (*p* = 0.007). Additionally, the mean impulse value measured by the OrthoLoad (713 Ns ± 108) was larger than AnyBody (463 Ns ± 139). The sEMG model showed a larger average impulse estimation (1007 Ns ± 484) than the OrthoLoad subjects, though this difference was not significant (*p* = 0.104).

## 4. Discussion

Our results suggest that the use of patient-specific trendlines to define sEMG-to-muscle force relationships reduced muscle force estimate error by ~5%, which corresponds to a potential error of 300 N when estimating L4/L5 compressive loading. In the case where less accuracy in estimation is acceptable, use of MVCs and rest trials alone can be used to define general relationships, saving clinical time.

Despite previous studies suggesting a difference in linearity between the extensor and flexor muscles, we failed to find a significant difference in error due to linearity alone for any muscle other than the LRA. The RRA and LEO showed an interactive effect between linearity and contraction speed. As contraction velocity increases, the muscle relationship of these muscles becomes more linear. One explanation may be that slower contraction speeds require additional muscle fiber recruitment to sustain the same force. As contraction speed increases, muscle force is sustained for a lesser time, requiring less recruitment, which is reflected in a linear instead of a logarithmic plot. An additional study controlling for sustained contraction time is necessary to study this effect.

Our EMG model showed a large variation in impulse and therefore compressive loading values. Variations in impulse can occur due to altering muscle force-to-sEMG relationships, individual fitness, differences from anthropomorphic data, and the amount of antagonist muscle activity. Antagonist muscle activity may also explain the larger impulse values observed by the sEMG model when compared with the AnyBody model, as the AnyBody model would not be affected by additional muscle force that may be generated when balancing agonist and antagonist muscle activity. This would be most apparent when subjects are standing upright, which would provide minimal loading from balancing momentum in the AnyBody model but might still have agonist and antagonist muscle activity. Despite the differences between the two models, both the EMG and AnyBody report compressive loading values found elsewhere in the literature [32]. This is consistent with our finding that the AnyBody model also reports lower loading than the OrthoLoad population. No difference was found between the population body weight being lifted by the OrthLoad population, making it unlikely that the lower AnyBody difference was due to differences in the study population.

Comparing the L4/5 results for our ball lifting task to Davenport et al. [31], we found slightly lower loads. Davenport et al. [31] found L5/S1 loads on the order of magnitude of six to ten times of body weight during an 11 kg knee-to-waist lift task. Our personalized EMG model predicted L4/5 peak loads from two to four-and-a-half times body weight but with a higher lumbar level, which is on par with their findings. Similarly, Banks et al. [33] recorded gait with 15% body weight dumbbells with an EMG model, including rectus abdominis, external obliques, and longissimus thoracis using an EMGopt software model and reported L4/5 loads 300% body weight, which again is in the same range for our reported surface EMG model.

The primary limitation of this study was the use of a small cohort to develop the sEMG model. While this may prevent the results of this study from being generalizable to large populations, it does highlight the potential advantage for using patient-specific sEMG models over generalizable models. Muscle cross-sectional areas and muscle moment arms were estimated via anthropometric data instead of actual measurement. Larger moment arms would decrease compressive load estimates by decreasing the amount of muscle force needed to balance moments around the L4/L5 joint. Cross-sectional muscle area was used to determine the proportion of force generation attributed to the EO or RA during flexion. This assumption could affect the calculated loading by misattributing force to the EO or RA. Overattributing force to the EO would result in higher loading as greater force would be needed to balance the moment generated from the EO compared to the RA. Misattribution of muscle force due to anthropomorphic assumptions similarly extends to the limited muscle selection of the study. Factoring additional muscles, especially those with a long moment arm relative to the spine, would substantially alter the lumbar load estimated by the EMG model. The inclusion of antagonist muscle activity would likely further increase the estimated lumbar load. We also would like to point to additional publications in the literature that use a small number of participants for model validation from six to ten participants [31,33,34,35,36,37].

Another limitation of this study was the lack of any method to factor in the effect of antagonist muscle activity. Brown et al. [27] reported that nonlinear muscle moments became linear when accounting for antagonist muscle activity, and that antagonist muscle activity could be as high as 298% of agonist muscle activity. Visually, our antagonist muscle activity never surpassed 50% of our agonist muscle activity, suggesting that accounting for antagonist muscle activity is less important for our model. The difference in observed antagonist activity may be due to differences in contraction activities, as Brown et al. [27] performed isokinetic contractions.

Previous studies of knee flexion have shown that isokinetic contractions have reduced antagonist activity when compared with isometric contractions. While it is possible this difference also exists between isokinetic and isometric contractions, to our knowledge, no such study searches for this difference. Finally, it is possible that the antagonist muscle activity was partially replaced by the resistance from the dynamometer lever arm. The inclusion of only six muscles meant that all flexion and extension moments had to be attributed to these few muscles. Previous studies have shown that trunk muscle activation can vary for different subjects performing the same task [38,39]. Since the extensor moment had to be fully attributed to the ES muscles, individuals who showed greater activation of the ES muscles would show abnormally high compressive loading values. This limitation, along with the muscle cross-sectional area and muscle moment differences, would alter muscle force estimations by a constant and would therefore change the ultimate compressive loading values, but not the shape of the muscle force-to-sEMG relationships.

## 5. Conclusions

We present exploratory work towards the development of a subject-specific model for measuring L4/L5 compressive loading for dynamic activity. While the EMG model was subject to high variability, loading values largely remained within those reported in the literature. Inclusion of additional muscles and antagonist activity would likely reduce the high variability between subjects and potentially change the shape of the muscle force-to-sEMG curve. Our EMG model suggests that further EMG model development should focus on subject-specific muscle force-to-sEMG relationships.

## Figures and Tables

**Table 1:** Summary of percent error values divided by measure factor.

Muscle	Specificity		Linearity		Contraction Speed		Significant Factors
	General	Specific	Linear	Logarithmic	30 deg/s	60 deg/s	
RES	22%	17%	17%	22%	18%	21%	Specificity
LES	23%	15%	17%	22%	18%	21%	Specificity
RRA	19%	16%	19%	16%	16%	19%	Specificity, linearity*speed, speed*
LRA	18%	14%	18%	13%	15%	17%	Specificity, linearity
REO	19%	15%	18%	16%	15%	19%	Specificity, linearity*speed, Specificity*linearity*speed*
LEO	18%	14%	18%	15%	14%	18%	Specificity

* indicates an interaction effect. deg/s, degrees per second; LEO, left external oblique; LES, left erector spinae; LRA, left rectus abdominus; REO, right external oblique; RES, right external oblique; RRA, right rectus abdominus.

## Data Availability

Data are available from the corresponding author upon request.

## Abbreviations

The following abbreviations are used in this manuscript:

sEMG	Surface electromyography
EMG	Electromyography
LBP	Low back pain
BMI	Body Mass Index
IVD	Intervertebral disk
LL	Lumbar lordosis
ES	Erector spinae
RA	Rectus abdominus
EO	External obliques
VCT	Voluntary contraction trials
SLSS	Straight-leg stoop-to-stand
RMS	Root mean square
RES	Right erector spinae
LES	Left erector spinae
RRA	Right rectus abdominus
LRA	Left rectus abdominus
REO	Right eternal oblique
LEO	Left external oblique
ANOVA	Analysis of variance
Deg/sec	Degrees per second
